# Instrumenting gait with an accelerometer: A system and algorithm examination

**DOI:** 10.1016/j.medengphy.2015.02.003

**Published:** 2015-04

**Authors:** A. Godfrey, S. Del Din, G. Barry, J.C. Mathers, L. Rochester

**Affiliations:** a Institute of Neuroscience, Newcastle University, Campus for Ageing & Vitality, Newcastle upon Tyne, UK; b Clinical Ageing Research Unit, Newcastle University, Campus for Ageing & Vitality, Newcastle upon Tyne, UK; c Institute of Cellular Medicine, Newcastle University, Campus for Ageing & Vitality, Newcastle upon Tyne, UK; d Human Nutrition Research Centre, Newcastle University, Campus for Ageing & Vitality, Newcastle upon Tyne, UK; e Department of Sport, Exercise and Rehabilitation, Northumbria University, Newcastle upon Tyne, UK

**Keywords:** Accelerometer, Validation, Gait, Algorithm, Healthy ageing

## Abstract

Gait is an important clinical assessment tool since changes in gait may reflect changes in general health. Measurement of gait is a complex process which has been restricted to the laboratory until relatively recently. The application of an inexpensive body worn sensor with appropriate gait algorithms (BWM) is an attractive alternative and offers the potential to assess gait in any setting. In this study we investigated the use of a low-cost BWM, compared to laboratory reference using a robust testing protocol in both younger and older adults. We observed that the BWM is a valid tool for estimating total step count and mean spatio-temporal gait characteristics however agreement for variability and asymmetry results was poor. We conducted a detailed investigation to explain the poor agreement between systems and determined it was due to inherent differences between the systems rather than inability of the sensor to measure the gait characteristics. The results highlight caution in the choice of reference system for validation studies. The BWM used in this study has the potential to gather longitudinal (real-world) spatio-temporal gait data that could be readily used in large lifestyle-based intervention studies, but further refinement of the algorithm(s) is required.

## Highlights

• Detailed investigation to explain poor variability/asymmetry agreement between accelerometers and instrumented walkway.
• Caution is urged in the choice of reference system for validation studies.
• Accelerometers have potential to gather continuous and robust spatio-temporal gait data, representative of normal living.
• Further refinement of the gait algorithms are required.

## 1 Introduction

Gait is a useful measure of overall health [1], and is a predictor for cognitive decline [2], falls status [3], quality of life [4] and longevity [5]. Thus, measuring characteristics of gait is becoming increasingly important as a robust method to determine many facets of health [6]. Typically, expensive (and large) laboratory systems, such as an instrumented walkway (e.g. GaitRite), are used to assess gait. While such a system is essential for developing and fine tuning protocols, its cost and size make it unviable to quantify gait characteristics in many settings [7]. This has driven the demand for cheaper and portable methods that can be more readily deployed, such as in large lifestyle-based intervention studies [6] allowing cost-effective and easy assessment of gait in a wide variety of environments [8].

As a result, the use of accelerometer-based body worn monitors (BWM, defined here as a sensor(s) with algorithms) and their application in instrumented testing has steadily risen in recent years [6,9–12]. Instrumented testing is not limited to any patient group, is not biased by age or gender differences and can provide highly accurate and objective data [7,13]. However, the popularity of BWM worn has been fuelled by commercial companies with black box methods of analysis and the introduction of a variety of accelerometer-based characteristics with little focus on which are the most valid [7,13–15]. Moreover, the closed system of analysis has created a limited understanding of the true strengths and weaknesses of algorithms.

Numerous testing limitations are also encountered within the literature. Typically, studies involving a BWM and instrumented walkway focus their attention on small (*N* = 7–23) single group sample sizes [16–18] making it difficult to considered the findings as representative of the groups. Robust testing of any BWM should include assessment of different populations (e.g. young/old [19–21]) and where homogeneity for gait characteristics may be low (healthy ageing), large sample sizes should be used to increase the ability to detect between group differences [22]. Alternatively, studies that have used larger sample sizes (*N* ≥ 80) have other limitations: a limited number of gait characteristics (3–5) with nondescript of age or pathology [15] or during a limited testing protocol [23]. These can be overcome by quantifying the appropriate mean, variability and asymmetry characteristics [1] during a suitable (continuous) testing protocol and separate estimates for left/right steps [24].

Our aim was to carry out a validation of a low-cost BWM to quantify a comprehensive group of gait characteristics in a large cohort of young and older adults to enhance generalisability, and to explore the sensitivity of the characteristics when comparing young and older adults. We adopted a suitable and robust methodology to examine a low cost BWM on the lower back during instrumented testing of gait in a large cohort of young and older adults to (i) define step count and quantify a comprehensive set of spatio-temporal gait characteristics described by the mean value, variability and asymmetry of each characteristic, (ii) compare the values to a laboratory reference and assess each system in gait quantification and (iii) compare discriminative gait characteristics of younger versus older adults by each system. We present our findings and discuss a new rationale for any poor agreement. The results from this study will help inform our ongoing work within the LiveWell Programme,^1^ defining a panel of measures which capture key features of healthy ageing during lifestyle-based intervention: the healthy ageing phenotype (HAP) [6].

## 2 Methods

### 2.1 Participant recruitment

Participants were recruited from staff and students at Newcastle University and VOICENorth,^2^ an older adult volunteer group who participate in research. Participants were included only if they were healthy i.e. had no physical or neurological disabilities that might impede their movement or balance. Eighty healthy adults aged 20–40 years (40 young healthy participants, YHP) and 50–70 years (40 older healthy participants, OHP) were recruited. All participants gave informed written consent and ethical consent for the project was granted by the National Research Ethics Service (County Durham and Tees Valley) and the Newcastle-upon-Tyne Hospitals NHS Foundation Trust (11/NE/0383).

### 2.2 Body worn monitor

**Fig. 1:**
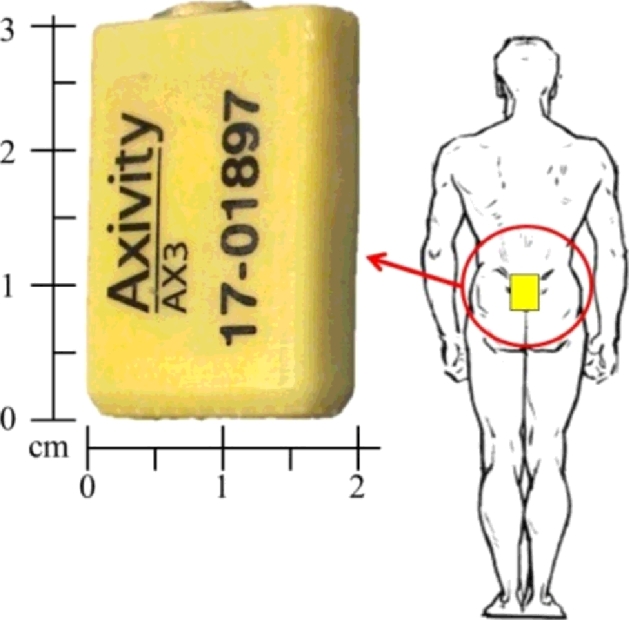
The accelerometer-based sensor and site of attachment on the lower back (L5).

Each participant wore a low cost (<£90) tri-axial accelerometer-based movement sensor^3^ (Fig. 1, dimensions: 23.0 mm × 32.5 mm × 7.6 mm, weight: 9 g) located on the fifth lumbar vertebrae (L5). The sensor was held in place by double sided tape and Hypafix.^4^ The sensor was programmed at a sampling frequency of 100 Hz (16-bit resolution) and at a range of ±8 g. Recorded signals were stored locally on the sensor's internal memory (512MB) as a raw binary file that was downloaded upon the completion of each participant trial.

### 2.3 Laboratory references

We used the GaitRite instrumented walkway and a video camera as the laboratory references for the gait characteristics in this study. The GaitRite dimensions were 7.0 m long and 0.6 m wide and had a spatial accuracy of 1.27 cm and sampling frequency of 240 Hz. Previous studies have verified that the GaitRite is a valid and reliable method for measuring mean gait characteristics in healthy younger and older adults [25]. During each walk, the video camera (Sony DCR-SR77) recorded at 25 frames per second and was used to determine total step count over the complete trial.

### 2.4 Experimental protocol and system set-up

**Fig. 2:**
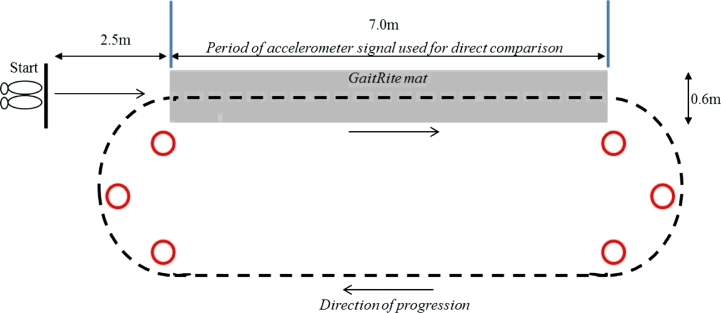
Pictorial representation of the walking route along the instrumented walkway (GaitRite) and around a 25 m loop.

Participants were instructed to perform a walking task under the condition of a normal, self-selected (preferred) walking pace. The walk was performed for 2 min and followed a 25 m route as illustrated in Fig. 2. This protocol was adopted based upon previous findings that the use of a continuous walking protocol of no fewer than 30 steps (≥50 steps optimal) is recommended when examining the reliability of gait variability [24]. In addition, the use of continuous walks limit any perturbations in the spatiotemporal rhythm of gait and the inflation of gait variability characteristics that are evident with repeated single trials [26].

The BWM was placed on L5 and could continuously gather data for the full test duration. However, GaitRite was placed in the circuit (Fig. 2) only allowing gait to be repeatedly sampled each time participants traversed the walkway [26,27]

### 2.5 Spatio-temporal characteristics: accelerometer algorithms

After testing was concluded, data were downloaded to a computer and analysed using a MATLAB^®^ program (R2012a). Temporal and spatial estimations of initial contact (IC), final contact (FC) and step length were derived from algorithms developed by McCamley et al. [28] and Zijlstra and Hof [29], respectively. These algorithms were designed for optimal use with a sensor on the lower back. A brief description of both is provided here.

#### 2.5.1 Temporal characteristics

**Fig. 3:**
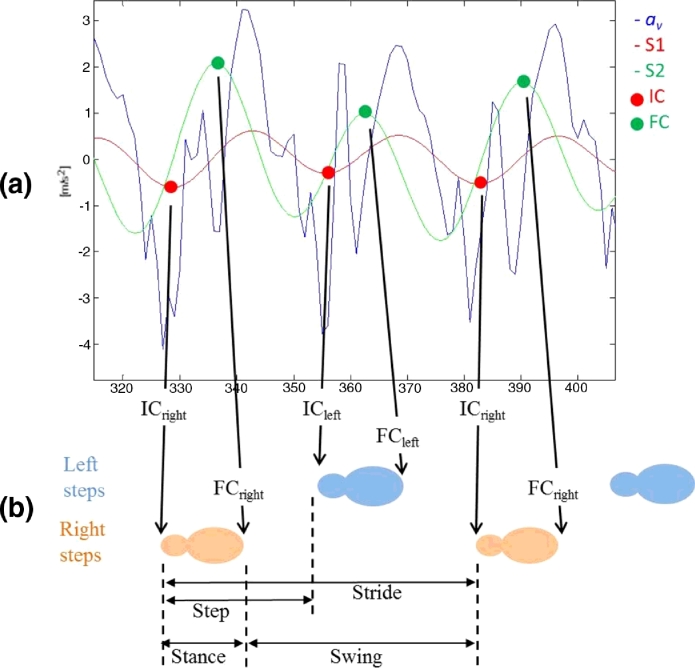
Calculation of step time, stance time, swing time and stride time from the detection of IC and FC events from left and right feet.

A continuous wavelet transform (CWT, convolution of the accelerometer data and an analysing function, i.e. mother wavelet) estimated IC/FC gait time events from the vertical acceleration (*a_v_*). Firstly *a_v_* was integrated and then differentiated using a Gaussian CWT, where IC's were identified as the times of the minima. The differentiated signal underwent a further CWT differentiation from which FC's were identified as the times of the maxima, Fig. 3(a). Initial inspection of the signal traces found spurious IC events (non-IC events which may constitute a scuff or artefact due to clothing). As a result, the algorithm was updated to include a previous methodology for step detection: restricting IC peaks within a predetermined timed interval (0.25–2.25 s) [30]. Whilst previous use of the algorithm estimated step time and stride time only, in this study we utilised the detection of IC/FC events for the novel estimation of stance time and swing time based upon the analysis of a gait cycle, Fig. 3(b).

Subsequently, the total number of steps estimated by the BWM was derived from the corrected algorithm. This was compared with the video recording for step count estimation. Additionally, the number of steps estimated by the corrected algorithm were used to segment the accelerometer data for direct comparison with GaitRite i.e. number of steps whilst on the GaitRite mat and in the remainder of the circuit. Previously, right and left ICs were identified by a gyroscope and the sign of the filtered vertical angular velocity at the instant of IC [28]. In comparison, the sensor used in this study had no gyroscope. Therefore right and left ICs were selected by the MATLAB program by using the number of manual observations (step counts and identification of first step as left or right) from the recorded video compared to BWM data. The accelerometer signal was segmented for direct comparison with the GaitRite based on number of steps on the walkway and number of steps around the remainder of the circuit back onto the walkway.

#### 2.5.2 Spatial characteristic

Step length was estimated from the up/downward movement of centre of mass (CoM). Movement in the vertical direction follows a circular trajectory during each single support phase; this is the inverted pendulum model [29]. If the changes in height (*h*) can be calculated (double integration of *a_v_*) step length can be predicted from Eq. (1) in which *l* refers to the pendulum length (height of the sensor from the ground to L5).

(1) $$\mathrm{step}\;\mathrm{length}=2\sqrt{2lh-h^{2}}$$

#### 2.5.3 Spatio-temporal characteristic

Step velocity was calculated from the simple relationship between (step) time and (step) length values, Eq. (2).

(2) $$\mathrm{step}\;\mathrm{velocity}=\frac{\mathrm{step}\;\mathrm{length}}{\mathrm{step}\;\mathrm{time}}$$

### 2.6 Gait characteristics: mean, variability and asymmetry

Data for individual steps were extracted from the GaitRite database using Microsoft Access.^5^ For both the GaitRite and BWM, mean gait values were calculated for step time, stride time, swing time, stance time, step length and step velocity, for left and right steps separately and then combined. For variability (standard deviation) and asymmetry values (Eq. (3)), left and right steps were calculated separately and then combined. The combined standard deviation of left and right steps was calculated by taking the square root of the mean variance of the left and right steps, Eq. (4). This method avoids confounding originating from asymmetry between left and right steps [24].

(3) $$\mathrm{Asymmetr}y_{\mathrm{left}\;\&\;\mathrm{right}}=\left|\mathrm{averag}e_{\mathrm{left}}-\mathrm{averag}e_{\mathrm{right}}\right|$$

(4) $$SD_{\mathrm{left}\;\&\;\mathrm{right}}=\sqrt{\frac{\mathrm{varianc}e_{\mathrm{left}\;\mathrm{steps}}+\mathrm{varianc}e_{\mathrm{right}\;\mathrm{steps}}}{2}}$$

### 2.7 Statistical analysis

Means and standard deviations (SD) were calculated for mean (BWM/GaitRite/video), variability and asymmetric data (both BWM/GaitRite) for both YHP and OHP. Normality of data distributions was tested with a Shapiro–Wilk test. Levels of agreement (LoA) between the laboratory references and BWM were expressed as intraclass correlation coefficients (ICCs) of type (2,*k*), mean differences between references and BWM ($\bar{x}$) ± 95% LoA and relative percentage. Pearson product–moment (*r*) and Spearman's rank (*ρ*) correlation coefficients were also calculated to measure the linear correlation (dependence) between laboratory references and the BWM.

Independent *t*-tests were used to examine the difference between groups and an analysis of covariance (ANCOVA) with height as a covariate to examine discriminative differences between groups (fixed factors) by each system. No Bonferroni correction was used due to the small number of comparisons (done per gait characteristic) between two groups (YHP/OHP or BWM/laboratory reference) [31]. For all analysis statistical significance was set at *p* < 0.05. Predefined acceptance ratings similar to previous recommendations for ICCs and LoA were set at excellent (>0.900, 0.0–4.9%), good (0.750–0.899, 5.0–9.9%), moderate (0.500–0.749, 10.0–49.9%) and poor (<0.500, >50.0%) [16,32].

## 3 Results

Eighty adults (40 YHP and 40 OHP) were recruited. Table 1 shows the characteristics of both groups. Within the OHP, three sensors failed to record leaving 37 participants for analysis. Although three individual data sets were lost during testing, this occurred during the initial phase of the study when the sensor was new to the market and some minor software problems were encountered. Upon correction of those issues by the manufacturer, the sensor was successfully deployed for the rest of the study.

### 3.1 System comparison

#### 3.1.1 Total step count

After applying the correction for spurious IC events, accuracy improved (YHP 13/40, OHP 7/37) but not all errors were eliminated. Table 2 presents the total number of continuous passes over the walkway, total step count and descriptive data for total number of steps accumulated. On average, both cohorts achieved the optimum number (≥50) of continuous steps for analysis with between five and nine passes over GaitRite. LoA between the video and BWM for total step count in both cohorts were excellent (ICCs between 0.969 and 0.986 for YHP, 0.938 and 1.000 for OHP, *p* < 0.05).

#### 3.1.2 Spatio-temporal characteristics: YHP

##### Mean

Table 3(a) shows positive mean differences ($\bar{x}$) and therefore greater (slower) estimates by the BWM in gait characteristic (5/6) estimation. Step, stride and stance time mean differences ±95% LoA and LoA (%) between the systems were excellent/good but slightly less so for length and velocity. Swing time had the poorest ICC results (<0.500) and only moderate LoA (%).

##### Variability

Table 3(c) shows ICCs for stride time and stance time were moderate but LoA (%) were poor. In general all other gait variability characteristics were poor.

##### Asymmetry

Table 3(e) shows ICCs, mean differences ±95% LoA and LoA (%) were poor for all asymmetric characteristics.

#### 3.1.3 Spatio-temporal characteristics: OHP

##### Mean

Similar to the YHP, Table 3(b) shows positive mean differences and therefore greater estimates by the BWM in gait characteristic (5/6) estimation. Step, stride and stance time mean differences ±95% LoA and LoA (%) between the systems were excellent/good but slightly less so for length and velocity. Swing time had good/moderate ICC and LoA (%) results.

##### Variability

Table 3(d) shows that stride time was the only characteristic where agreement and correlation was good between systems (ICC = 0.886). All other characteristics were poor.

##### Asymmetry

Table 3(f) shows ICCs, mean differences ±95% LoA and LoA (%) were poor for all asymmetric characteristics though ICC for swing time was moderate.

### 3.2 Discriminative analysis for age: BWM versus GaitRite

Table 3 also shows the discriminative analysis of YHP versus OHP between the GaitRite and the BWM based on the ANCOVA (values in bold) with participant height as a covariate. Neither of the systems agreed on between group discrimination for any of the estimated gait characteristics. Where between group differences were observed, the GaitRite found differences for variability of swing time (*p* = 0.002) and step length (*p* = 0.006). The BWM found significant differences between groups for mean step length estimation (*p* = 0.023). Marginally significant differences were observed with the BWM for mean step velocity (*p* = 0.058) and asymmetry of step time (*p* = 0.054).

## 4 Discussion

To our knowledge this is the first study to take a comprehensive approach to quantify gait characterising 17 different features of gait. Furthermore this is one of the largest studies to date to compare YHP and OHP with a comprehensive set of gait characteristics during a suitable protocol, allowing greater confidence in the generalisability of findings. The key findings from this study found excellent agreement for total step count and mean values; however, agreement for variability and asymmetry was generally poor. Both systems were able to discriminate with respect to age; however, the characteristics were different. The ability to accurately and confidently replicate gait characteristics using a low-cost BWM has significant implications in large lifestyle-based intervention studies where cost and testing environment pay a key role in determining measures to study the intervention effect [6].

### 4.1 Algorithm performance: step count and spatio-temporal gait characteristics

The algorithms used in this study to estimate IC/FC gait events [28] and step length [29] have been used previously with small numbers of younger and older adults, but this is their first combined use in a large study of two contrasting age groups with a suitable protocol. This study is also the first to examine the IC/FC algorithm in older adults (≥50 years) and to utilise those events to estimate step count, stance time, swing time and step velocity. While the algorithm resulted in excellent/good agreement for step count and mean step and stride times, results were good/moderate for stance and swing times. A fundamental explanation for this is how the FC events are derived from vertical acceleration where the wavelet transform operation of smoothing (integrating) and double differentiation, while powerful, can inhibit both resolution and signal to noise ratio based on wavelet selection [33]. One possible method for improving the FC event estimations is the alternative use of mother wavelet where it has been suggested that a bi-orthogonal spine wavelet is superior to that used here, i.e. Gaussian [33]. An additional benefit of alternate wavelet selection may be the elimination of the spurious IC peaks. Although we introduced a restriction on IC events based upon previous findings [30], not all spurious events were eliminated. The use of more suitable wavelet techniques may improve IC/FC detection and subsequently stance and swing times as both are estimated from the IC/FC sequence of events within the gait cycle, Fig. 3(b).

### 4.2 System comparison, BWM versus video: step count

We utilised the IC/FC algorithm with great effect to estimate the total steps walked by all the participants. The algorithm has not been used previously for step count estimation and ICC results were excellent for both groups. One pleasing aspect of this result is the estimation of total steps during continuous walking incorporating linear and curvilinear trajectories. Previous step count estimation by a BWM has been assessed during short straight line walking or on a treadmill, protocols which fail to capture habitual walking habits [34,35]. Accurate step count estimation can play an important role in lifestyle-based interventions where this simple outcome can inform long-term trial effectiveness [36] or public health recommendations [37] where older adults often fail to meet basic physical activity guidelines [38]. Additionally, accurate step count estimation can be used in a more abstract (pattern) analysis examining stepping ranges, offering new and simple insights [39].

### 4.3 System comparison, BWM versus GaitRite: average, variability and asymmetry

We quantified six spatio-temporal gait characteristics in YHP and OHP, as age is known to influence gait characteristics [21], and found excellent/good agreement for average values that were within acceptable ranges: GaitRite/BWM characteristics in YHP were comparable to similar studies [15,40–42] for step (referenced data in italics versus our results: *0.55/0.54 s* versus 0.53/0.54 s), stride (*1.10/1.08 s* versus 1.07/1.07), stance (*0.65/0.70 s* versus 0.67/0.71) and swing (*0.45/0.42 s* versus 0.40/0.37) times as well as step length (*77.83/80.00 cm* versus 75.86/78.63 cm) and velocity (*142.49/154.75 cm/s* versus 142.99/147.78). OHP values were also comparable to other studies [1,15,41,43] for: step (*0.51/0.52 s* versus 0.52/0.52 s), stride (*1.12/1.05 s* versus 1.04/1.05 s), stance (*0.69* *s* versus 0.65/0.68 s) and swing (*0.39* *s* versus 0.39/0.37 s) times but less so for step length (*67.00/73.75 cm* versus 72.94/79.83 cm) and velocity (*135.00/140.00 cm/s* versus 141.08/153.70 cm/s). Any difference of note is observed in the spatial characteristics for OHP which can be attributed to difference in cohort ages (mean 71.3 years for referenced studies and therefore a reduced step length/ velocity) for GaitRite. Further differences can be attributed to sensor placement (*L3* versus L5) where the inverted pendulum model is prone to length deviations due to dependence on straight line walking [29] and length of pendulum (sensor height) due to a generic correction factor [29] that may/may not have been applied. The underestimation of swing time by the BWM compared with GaitRite in YHP is also seen in other studies (reference data shown previously in italics) but we found no published estimates of accelerometer derived swing time in OHP to compare with our data. Examining the most commonly reported characteristics (step and stride times), we observe that agreement for stride time is mostly higher due to the combination of left/right steps within a stride, a direct result of the confounding effect of limb asymmetry [44].

Similar to previous studies, we found poor agreement between systems for estimates of variability and asymmetry [15,16,41,45] but estimates of stride time variability in the OHP were in good agreement. However, direct comparison of variability and asymmetric results was difficult because the referenced studies presented their results as coefficient of variation (%) and the difference between left and right steps divided by the bilateral average, respectively. Plausible explanations for the differences between the systems, e.g. drift due to integration, which are also applicable to this study, have been reported previously [16,46]. While great care was taken to adopt the most suitable protocol and, therefore, to minimise confounding factors, the variability and asymmetry differences between the two systems may be a result of the two curvilinear segments of the circuit participants were asked to walk. Analysis of video recordings showed that some participants did not always maintain a uniform walking pattern when walking i.e. some participants turned abruptly when rounding the ends of the track rather than maintaining a uniform curvilinear path. These abrupt changes in gait (more varied spatio-temporal characteristics) may have been included during the automated segmentation of the BWM data for direct comparison to GaitRite.

**Fig. 4:**
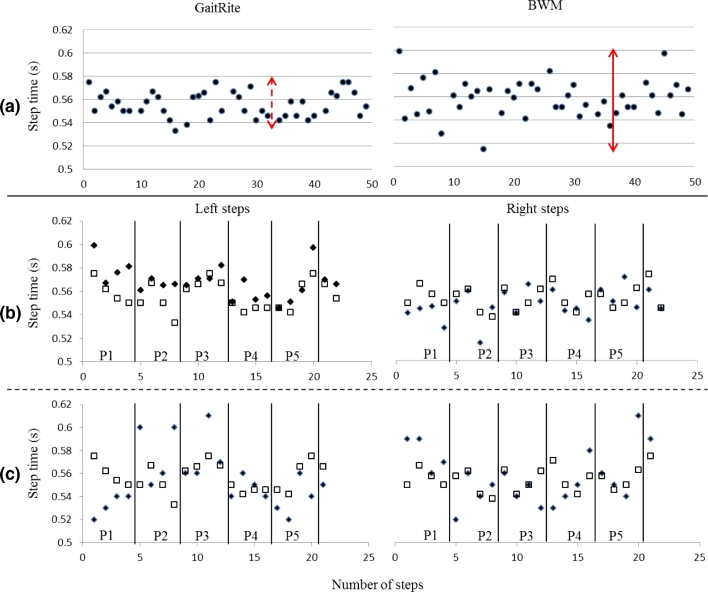
Example of YHP step time data as quantified by the GaitRite (white square) and BWM (black diamonds): (a) all steps, arrows indicate the greater variability of estimated step times for the GaitRite (small) and BWM (large); (b) correct allocation of left steps and right steps, some step times are closely matched (good agreement and correlations); (c) an example of where incorrect allocation of left and right steps may have occurred (poor agreement and correlation), e.g. P1 of left step. P1–P5 indicate the numerous passes the YHP completed over the GaitRite.

Additionally, manually observed steps and progression to the next pass on the GaitRite rather than direct system synchronisation were used to segment the accelerometer data via MATLAB. As can be seen from Table 2, the agreement between the two systems was excellent but not exact (mean difference: 1–4 steps). The slight difference in number of steps and subsequently the absolute distinction between left and right for some passes over the GaitRite were not identical for incrementing passes. This could have led to potential errors in the identification of left and right steps (mixing of both), i.e. MATLAB assumed consecutive left/right steps and segmented according to the count based on accelerometer data where ±1 or three step(s) would lead to incorrect comparison to GaitRite. As a result this had a negative impact on variability and asymmetry results, i.e. low agreement. To further investigate this error and to explore the technical quantification of gait by both systems, we selected step time as a probing characteristic. We plotted the step times as quantified by both systems and found that while the BWM accurately identified each step it did so with a greater range of values, Fig. 4(a). This accounted for the higher BWM variability of step time (Table 3). Subsequently, low agreement in variability and asymmetry can be accounted for with the correct and mixed allocation of left/right steps in Fig. 4(b) and (c), respectively, by the MATLAB program.

### 4.4 System comparison, BWM versus GaitRite: discriminating ageing cohorts

Neither the BWM nor GaitRite was better in distinguishing age groups. Previous research involving a walkway for the distinguishing between gait characteristics of younger and older adults found the variability of step width as the more sensitive gait characteristic but this approach was not possible in our study due to the limitation of a single tri-axial accelerometer [47]. However, we found significant differences between groups in GaitRite data for step length variability, which is similar to another study using treadmill testing [48]. We did not find published data against which to compare our observations of a significant between group difference in swing time. Moreover, distinction between groups with mean step length data with a BWM was not available from the literature. Previously, where average gait characteristics were non-discriminatory between groups, more complex methods of analysis (repeating pattern) have proved useful [49]. However, those repeating patterns become evident only during prolonged periods of testing (1 h) [50].

### 4.5 System comparison, BWM versus GaitRite: technical differences

Though the GaitRite system has been used effectively for gait quantification, its functioning (pressure sensing) is unrelated to the workings of a BWM (accelerations). Therefore, a walkway is not the most suitable laboratory reference to ‘validate’ a BWM. The primary purpose of a BWM (worn on L5) is to *continuously* track the body resulting in a constant signal that is representative of whole body movement, compared with the intermittent foot falls on a walkway (Fig. 3). The resulting peak(s) therefore represents the trajectory of the CoM rather than the true heel strike (IC) or toe off (FC) events determined by GaitRite. The IC/FC events detected for the purposes of this study (or any study) are more accurately described as best estimates due to the sensor location and biomechanical properties of the musculoskeletal segments.

Previous work using continuous tracking of the CoM with 3D motion analysis supports this suggestion [51,52]. Direct comparison of vertical acceleration, velocity, and position traces together with spatial—temporal parameters showed good agreement between an optical motion capture system and a BWM. What is clear is that even the comparison of a BWM to a 3D system, though more similar in their quantification of gait, measure different components; acceleration and displacement, respectively. Though acceleration and displacement can be related through single and double integration/derivation, that process introduces error through drift, where the error in the signal after each integration increases by *ε* = *t*^1.5^, where *t* is integration time and *ε* is error [51,53].

Furthermore, scuffs can be detected by GaitRite before a true heel strike or IC event has occurred due to the casual walking patterns of some participants [54], which were also observed during our testing. Those phenomena required the manual processing of GaitRite data to eliminate scuff events, i.e. removal of random points of contact/pressure on the walkway. This subjective inclusion/exclusion of contact areas on the walkway can be a further source of discrepancy between spatio-temporal data acquired by pressure sensors directly under foot and representation of a whole body motion through space. With a spatial accuracy of 1.27 cm the inclusion/exclusion of a contact area can be the difference of approximately 0.009 s based on estimated stepping speed (step velocity of 142 cm/s).

Moreover, the algorithms are dependent on the signal characteristics e.g. peak detection methods reliant on polynomial coefficients or local maxima to locate the maximum/minimum. Any delay in the location of the maximum (due to smoothness of peak) as a result of filtering or processing methods can introduce timing differences between the BWM and GaitRite. These algorithm and spatial GaitRite differences in estimated times at the millisecond level have a negative impact on agreement between systems where the resolution of temporal characteristics is quantified, Table 3 and Fig. 4.

## 5 Conclusion

The accelerometer-based sensor and algorithms used in this study form a useful BWM (worn on the lower back) for the purposes of instrumenting gait in healthy adults. Step count and mean spatial—temporal characteristics had excellent/good agreement with laboratory references during a protocol representative of prolonged habitual walking. In contrast, there was poor agreement between methods for estimates of left/right step data, variability and asymmetry. We conducted a detailed investigation to explain the poor agreement between systems and determined it was due to inherent differences between the systems rather than inability of the sensor to measure the gait characteristics. The results highlight caution in the choice of reference system for validation studies. Neither approach was better at distinguishing between gait characteristics of younger and older groups of healthy adults. However, due to its functionality, the BWM used here has the potential to gather continuous and robust spatio-temporal gait characteristics more representative of normal living, offering the opportunity to use novel analysis (fractals/patterns) to extract additional information. Further refinement of algorithms is recommended to optimise BWM applicability.

## Conflict of interest

There is no conflict of interest.

## Funding

J.C.M. leads, L.R. is an investigator on and A.G. was supported by LiveWell a research project funded through a collaborative grant from the Lifelong Health and Wellbeing (LLHW) initiative, managed by the Medical Research Council (MRC) on behalf of the funders: Biotechnology and Biological Sciences Research Council, Engineering and Physical Sciences Research Council, Economic and Social Research Council, Medical Research Council, Chief Scientist Office of the Scottish Government Health Directorates, National Institute for Health Research (NIHR)/The Department of Health, The Health and Social Care Research & Development of the Public Health Agency (Northern Ireland), and Wales Office of Research and Development for Health and Social Care and the Welsh Assembly Government (grant number: G0900686). L.R. and A.G. are supported by the National Institute for Health Research (NIHR) Newcastle Biomedical Research Centre and Unit based at Newcastle upon Tyne Hospitals NHS Foundation Trust and Newcastle University. The views expressed are those of the authors and not necessarily those of the NHS or NIHR or the Department of Health. S.D.D. and G.B. are supported by the V-Time project, which is a European Union 7th Framework Programme (FP7) under the health theme (FP7: 278169).

## Ethical approval

All participants gave informed written consent and ethical consent for the project was granted by the National Research Ethics Service (County Durham and Tees Valley) and the Newcastle-upon-Tyne Hospitals NHS Foundation Trust (REC reference 11/NE/0383).

## Figures and Tables

**Table 1:** Demographical details on the YHP and OHP cohorts.

	YHP (*n* = 40)	OHP (*n* = 37)	*p*
Age (years)	28.62 ± 5.32	63.78 ± 6.40	0.000
Height (cm)	172.26 ± 8.75	165.86 ± 9.26	0.002
Weight (kg)	72.83 ± 13.71	70.87 ± 14.84	0.544

**Table 2:** Average number of steps on and passes (walks) over the GaitRite for each group at each walking speed during 2 min with correlations between total step count for the accelerometer and reference (video).

Group	Task	Video	Accelerometer	ICC	*R*
				
		Mean ± SD			
YHP	Steps on GaitRite	66 ± 5		0.969	0.964
	Passes over GaitRite	7 ± 1			
	Total steps	238 ± 20	234 ± 19		
OHP	Steps on GaitRite	67 ± 8		1.000	0.969
	Passes over GaitRite	7 ± 1			
	Total steps	243 ± 27	246 ± 21		

**Table 3:** ICC, correlations, mean difference ($\bar{x}$) ± 95% LoA and LoA% between the BWM and GaitRite for mean/variability/asymmetry step time, stride time, stance time, swing time, step length and step velocity for both cohorts.

Group	Task	Mean ± SD		Correlations/agreement				
		GaitRite	BWM	ICC	*r*	*ρ*	$\bar{x}\pm 95\%$	LoA (%)
*Mean*								
(a) YHP	Step time (s)	0.534 ± 0.038	0.535 ± 0.039	0.997*	0.994*	0.991*	0.002 ± 0.005	1.0
	Stride time (s)	1.070 ± 0.077	1.072 ± 0.076	0.998*	0.997*	0.987*	0.003 ± 0.012	1.1
	Stance time (s)	0.668 ± 0.056	0.707 ± 0.057	0.845*	0.904*	0.895*	0.039 ± 0.049	7.1
	Swing time (s)	0.401 ± 0.026	0.365 ± 0.029	0.487*	0.591*	0.592*	–0.035 ± 0.049	12.8
	Step length (cm)	75.862 ± 5.793	**78.630 ± 9.301**^a^	0.828*	0.833*	0.829*	2.770 ± 10.811	14.0
	Step velocity (cm/s)	142.988 ± 14.494	147.783 ± 19.086	0.901*	0.882*	0.860*	4.795 ± 18.199	12.5
(b) OHP	Step time	0.519 ± 0.032	0.522 ± 0.034	0.997*	0.997*	0.992*	0.003 ± 0.003	0.5
	Stride time	1.039 ± 0.064	1.045 ± 0.065	0.999*	1.000*	0.999*	0.004 ± 0.004	0.4
	Stance time	0.652 ± 0.047	0.679 ± 0.042	0.877*	0.918*	0.897*	0.026 ± 0.037	5.6
	Swing time	0.387 ± 0.023	0.365 ± 0.028	0.701*	0.740*	0.756*	−0.022 ± 0.037	9.8
	Step length	72.942 ± 7.319	**79.828 ± 9.797**^a^	0.790*	0.880*	0.831*	6.803 ± 9.377	12.3
	Step velocity	141.078 ± 14.540	153.701 ± 20.299	0.815*	0.900*	0.867*	12.664 ± 18.541	12.6
*Variability*								
(c) YHP	Step time	0.013 ± 0.011	0.019 ± 0.009	0.109	0.067	0.248	0.005 ± 0.026	161.2
	Stride time	0.018 ± 0.005	0.023 ± 0.007	0.549**	0.534*	0.600*	0.005 ± 0.012	59.8
	Stance time	0.015 ± 0.004	0.022 ± 0.009	0.428**	0.546*	0.530*	0.008 ± 0.015	80.1
	Swing time	**0.010 ± 0.002**^b^	0.021 ± 0.011	0.067	0.176	0.330**	0.010 ± 0.022	137.8
	Step length	**1.782 ± 0.426**^c^	4.859 ± 1.908	−0.015	−0.062	0.099	3.077 ± 3.882	116.9
	Step velocity	4.883 ± 1.147	10.548 ± 3.759	−0.012	−0.032	0.127	5.686 ± 7.772	100.6
(d) OHP	Step time	0.013 ± 0.005	0.019 ± 0.012	0.353	0.401**	0.289	0.006 ± 0.020	125.4
	Stride time	0.019 ± 0.006	0.022 ± 0.006	0.886*	0.854*	0.807*	0.002 ± 0.006	31.2
	Stance time	0.014 ± 0.004	0.020 ± 0.007	0.365**	0.413**	0.467**	0.006 ± 0.013	73.7
	Swing time	**0.012 ± 0.003**^b^	0.017 ± 0.007	0.468**	0.519**	0.436**	0.005 ± 0.012	83.1
	Step length	**2.046 ± 0.579**^c^	4.792 ± 1.858	0.060	0.162	0.186	2.746 ± 3.636	106.3
	Step velocity	4.841 ± 1.198	10.816 ± 4.045	0.039	0.108	0.119	5.973 ± 8.023	102.5
*Asymmetry*								
(e) YHP	Step time	0.006 ± 0.007	0.008 ± 0.017	0.129	0.096	−0.010	0.002 ± 0.031	407.4
	Stride time	0.002 ± 0.002	0.003 ± 0.002	−0.056	−0.027	−0.085	0.000 ± 0.006	239.1
	Stance time	0.006 ± 0.005	0.009 ± 0.015	0.040	0.036	0.090	0.003 ± 0.030	397.6
	Swing time	0.006 ± 0.004	0.009 ± 0.016	0.063	0.063	−0.005	0.003 ± 0.032	410.9
	Step length	1.756 ± 1.274	1.324 ± 1.753	0.275	0.171	0.200	−0.422 ± 3.890	253.5
	Step velocity	3.464 ± 2.758	3.688 ± 5.862	0.444**	0.364**	−0.093	0.223 ± 10.770	301.2
(f) OHP	Step time	0.009 ± 0.008	0.011 ± 0.012	0.381	0.265	0.094	0.004 ± 0.020	210.5
	Stride time	0.002 ± 0.002	0.002 ± 0.002	0.333	0.201	−0.039	0.000 ± 0.005	221.2
	Stance time	0.007 ± 0.006	0.009 ± 0.008	0.439**	0.301	0.037	0.002 ± 0.016	204.8
	Swing time	0.007 ± 0.006	0.009 ± 0.008	0.545**	0.396**	0.144	0.002 ± 0.016	195.4
	Step length	1.641 ± 1.407	1.505 ± 1.222	−0.189	−0.085	0.000	−0.135 ± 3.804	241.9
	Step velocity	3.468 ± 3.507	3.570 ± 3.849	−0.394	−0.160	−0.087	0.102 ± 10.990	312.3

a,b,b Significant difference between BWM and GaitRite for corresponding gait characteristics.

* *p* < 0.001.

** *p* < 0.05.

## Footnotes

1 LiveWell is a research programme intended to develop interventions to enhance health and well-being in later life. LiveWell focusses on the retirement period (55–70 years) as a window of opportunity for successful intervention, http://www.livewell.ac.uk.

2 www.ncl.ac.uk/changingage/engagement/VOICENorth.

3 Axivity AX3, York, UK. This is a movement sensor and not specifically designed for gait instrumentation.

4 BSN Medical Limited, Hull, UK.

5 Microsoft Corp., Redmond, WA, USA.
